# Enhanced Foamability with Shrinking Microfibers in Linear Polymer

**DOI:** 10.3390/polym11020211

**Published:** 2019-01-26

**Authors:** Eric S. Kim, Heon E. Park, Carlos R. Lopez-Barron, Patrick C. Lee

**Affiliations:** 1 Department of Mechanical and Industrial Engineering, University of Toronto, 5 King’s College Road, Toronto, ON M5S 3G8, Canada; ericskim@mie.utoronto.ca; 2 Department of Mechanical Engineering, University of Vermont, 33 Colchester Avenue, Burlington, VT 05405, USA; 3 ExxonMobil Chemical Company, Baytown Technology and Engineering Complex, Baytown, TX 77520, USA; carlos.r.lopez-barron@exxonmobil.com

**Keywords:** strain hardening, polymeric foaming, in situ shrinking microfiber

## Abstract

Strain hardening has important roles in understanding material structures and polymer processing methods, such as foaming, film forming, and fiber extruding. A common method to improve strain hardening behavior is to chemically branch polymer structures, which is costly, thus preventing users from controlling the degree of behavior. A smart microfiber blending technology, however, would allow cost-efficient tuning of the degree of strain hardening. In this study, we investigated the effects of compounding polymers with microfibers for both shear and extensional rheological behaviors and characteristics and thus for the final foam morphologies formed by batch physical foaming with carbon dioxide. Extensional rheometry showed that compounding of in situ shrinking microfibers significantly enhanced strain hardening compared to compounding of nonshrinking microfibers. Shear rheometry with linear viscoelastic data showed a greater increase in both the loss and storage modulus in composites with shrinking microfibers than in those with nonshrinking microfibers at low frequencies. The batch physical foaming results demonstrated a greater increase in the cell population density and expansion ratio with in situ shrinking microfibers than with nonshrinking microfibers. The enhancement due to the shrinkage of compounded microfibers decreasing with temperature implies that the strain hardening can be tailored by changing processing conditions.

## 1. Introduction

The strain hardening behavior of molten polymers has important roles in characterization and manufacturing, which is demonstrated by the tensile stress growth coefficient above the linear (invariant with rate) curve during extensional flow. This nonlinear behavior can promote a self-healing effect [1], leading to more homogenous deformation and less flow instability during extensional flow. Thus, we can expect that the strain hardening behavior in polymeric materials is an indicator to determine the processability of manufacturing areas that involves strong extensional flow, such as foaming, film blowing, and fiber spinning. Regarding the thermoplastic foaming process, a higher degree of strain hardening under extension can increase the cell population density by reducing cell coalescence [2,3,4,5].

Chemically introducing long-chain branching on linear polymeric chains has been a standard method to improve strain hardening and has been studied extensively [6,7,8]. However, efforts to produce polymers with controlled branching structure to achieve the desired strain hardening are still quite costly, and once a long-chain branched polymer has been synthesized, changing the behavior becomes difficult. Even though various methods have been studied to improve the properties and processability of polymers [2,9,10,11], few published studies exploit the compounding of fiber materials with shrinking characteristics with a matrix material to change the physical characteristics of the matrix and eventually obtain the desired properties of the products, such as the tailored foamability of polymers.

**Figure 1:**
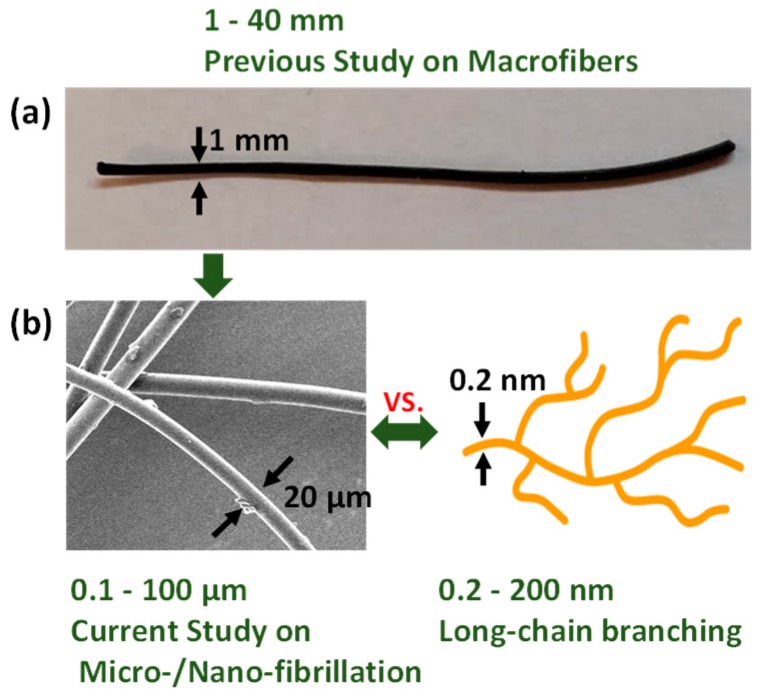
Comparison among fibers used in the previous and current studies, along with long-chain branching. (**a**) The previous study [12] was conducted using macroscale shrinking fibers, which showed the feasibility of the improvement of strain hardening as well as the foamability. (**b**) As a next phase, the current study has been conducted with microscale fibers.

In our previous study [12], we pioneered the technology to enhance and control strain hardening of linear polymers by compounding with heat-shrinking fibers to tailor the resulting properties by changing certain operating conditions, such as temperature. While the shrinkage of fibers outside of the matrix occurs upon activation due to a change in temperature, the shrinkage of fibers in the matrix is suppressed by the properties of the matrix, causing the fibers to generate compressive stress in the matrix. Our current study aims to present a polymeric composite reinforced by microscale in situ shrinking fibers to more precisely control the strain hardening behavior (Figure 1).

In this study, we hypothesized that compounding a linear polymer matrix with shrinking “microfibers” would show a more apparent enhancement of strain hardening upon extensional deformation and that such enhancement could be more accurately controlled by changing the operating temperature than in our previous work. We chose polypropylene (PP) as a matrix material because PP is one of the most popular polymers due to its unique combination of desirable chemical and physical properties. For example, PP has a high temperature resistance/stiffness, high impact strength, and great chemical resistance [13]. However, conventional linear PP has a relatively low melt strength and shows no strain hardening in extensional flow, which together limit the processing window considerably [14,15]. To investigate the effect of shrinkage, extensional viscosity measurements were described in terms of strain hardening behaviors in PP composites containing shrinkable or heat-activated shrinking (HAS) microfibers (PP/HAS). PP/HAS was compared to those containing preshrunk or heat-passive (HP) microfibers (PP/HP) and neat PP. Microfibers were also fabricated from PP, which is beneficial for recycling [16]. The single microfiber tensile test for the two types of microfibers was conducted to prove that the improved strain hardening behavior resulted from the shrinking mechanism. Microfiber pull-out tests were conducted to reinforce our hypothesis by confirming the existence of adhesion between the matrix and the microfibers. Extensional rheometry was conducted to show strain hardening due to shrinking microfibers and to study the possibility of controlling the degree of strain hardening using the processing temperature. Shear rheometry was performed to investigate the resemblance with cylinder-type microphase separation in block copolymers. Foams were generated using carbon dioxide (CO_2_) as a physical blowing agent to demonstrate the effect of enhanced strain hardening on the properties of the foams, such as cell population density and volume expansion ratio.

## 2. Materials and Methods

### 2.1. Materials

We used a random linear propylene–ethylene copolymer (e-PP) received from A. Schulman, Inc. (DS6D82, Fairlawn, Ohio, United States) as a matrix. We also chose another PP material to fabricate microfibers and anticipated recycling without issue. We fabricated microfibers using isotactic PP (i-PP) purchased from Sigma-Aldrich (Product number 182389, St. Louis, MO, USA). We carefully chose those two PPs to achieve a difference in melting points large enough to characterize and process when the matrix is molten and the microfibers are not, therefore retaining the fiber structure and having a sufficient temperature window to study the possibility of tailoring the strain hardening. The shrinking activation temperatures with/without dissolved CO_2_ were within the processing temperature window (i.e., between two melting points of the e-PP and i-PP). CO_2_, as a physical blowing agent, was purchased from Airgas with purity over 99%.

### 2.2. Sample Preparation

**Figure 2:**
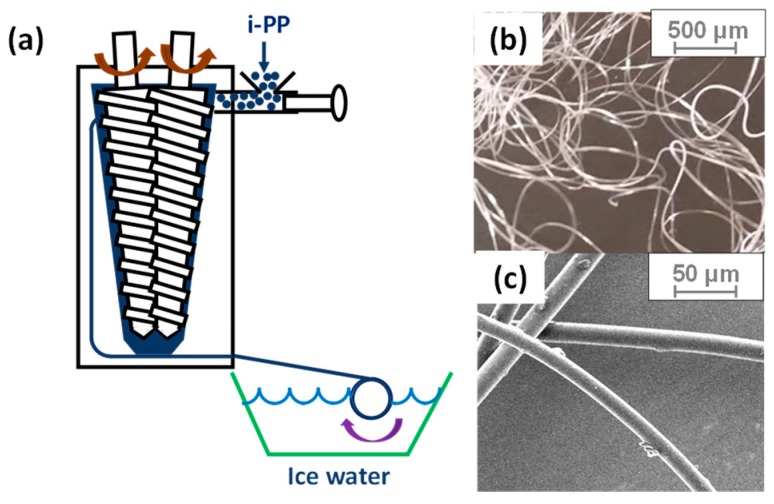
(**a**) Schematics of a fiber spinning system to fabricate heat-activated shrinking (HAS) microfibers. The heat-passive (HP) microfibers were fabricated without icy water and were cooled at room temperature while they are continuously collected. (**b**,**c**) Microfibers fabricated by fiber spinning.

All the resins were dried in a vacuum oven at room temperature for 24 h before preparation. Microfibers of i-PP were fabricated by spinning with a twin-screw compounder (DACA Instruments, Santa Barbara, CA, USA), as shown in Figure 2. The screw speed was set to 25 rpm, while the barrel temperature was maintained at 240 °C. To create two different types (HAS and HP) of microfibers, we cooled down the microfibers after spinning under two different conditions: quenching with icy water to make shrinkable (HAS) fibers that will shrink during operation and leaving at room temperature to make shrunk (HP) microfibers that will no longer shrink. The microfibers had an average diameter of 25 μm and were cut to 18 mm in length. Thus, the aspect ratio was approximately 720.

**Figure 3:**
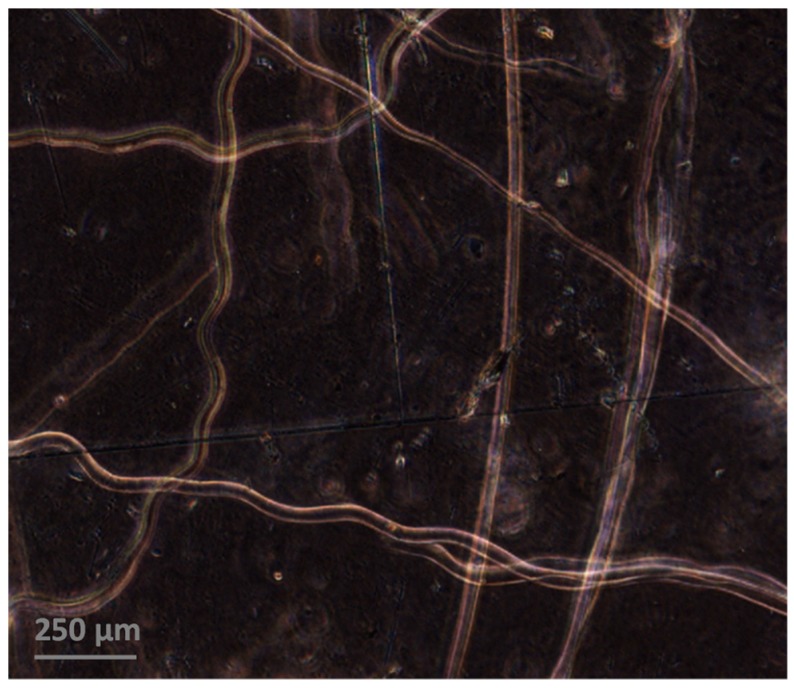
Morphology of e-PP/R-HAS microfibers. The microfibers were well dispersed in the e-PP matrix. The microfibers also showed a uniform diameter (25 ± 4 μm).

**Figure 4:**
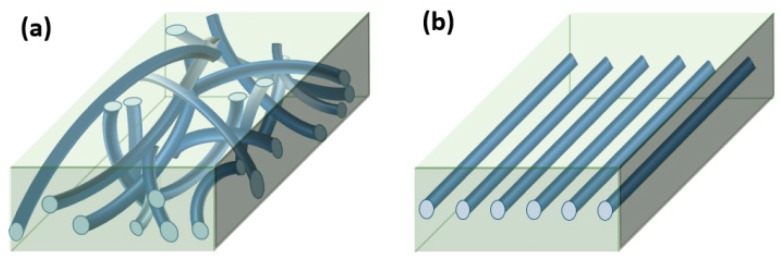
Specimens for testing, (**a**) e-PP/R-HAS and e-PP/randomly distributed HP (R-HP) microfibers for extensional/shear rheometry and foaming and (**b**) e-PP/linearly distributed HAS (L-HAS) and e-PP/linearly distributed HP (L-HP) microfibers along the extensional axis for extensional rheometry. The microfibers are drawn larger than their actual sizes. (Color should be used).

We tested samples with two different ways of aligning the microfibers in the matrix. First, to prepare e-PP/randomly distributed microfibers (e-PP/R-HAS, e-PP/R-HP) for extensional/shear rheometry and foaming, dried e-PP and the microfibers were compounded using a 2-piece roller blade mixer (CW Brabender, South Hackensack, New Jersey, United States) at the screw speed of 15 rpm, at 150 °C, which is lower than the melting point of the microfibers and higher than that of the e-matrix. The morphology of the composite, shown in Figure 3, was examined using a digital inverted microscope (Fisher Scientific, Hampton, NH, USA). The resultant composite showed well-dispersed microfibers with uniform diameters. The composites were reshaped using compression molding (Carver Inc., Wabash, IN, USA), with a rectangular mold for extensional rheometry and a circular mold for shear rheometry, and batch foaming experiments. The dimensions of each mold were 18 mm × 10 mm × 0.7 mm and 25 mm in diameter with 3 mm height, respectively. Figure 4a shows a schematic of these samples. All samples were made with 4 wt % (4.2 vol %) microfibers.

To prepare e-PP/linearly distributed microfibers (e-PP/L-HAS, e-PP/L-HP) for extensional rheometry, microfibers were aligned in the extensional direction on top of a 1.5-mm-thick slab of e-PP, and another slab was placed on top of those. Then, the set was compression-molded at 150 °C to achieve the sample shown in Figure 4b.

### 2.3. Microfiber Shrinkage Ratio

We determined the shrinkage ratio of the HAS microfibers at various pressures with/without dissolved CO_2_. The HAS microfibers were cut to 10 mm. A PTFE film was layered on the bottom of a chamber. This film was to prevent the heated microfibers from being physically contacted to the oven surface, causing resistance during shrinkage. The chamber was heated to set temperature. Then, the microfibers were placed in the heated chamber and taken out after 5 min without applying CO_2_ for the 1 atm test or with applied CO_2_ for high-pressure tests. The time of 5 min was selected because it was sufficient time for microfibers to complete the shrinkage, and further shrinkage did not occur after 5 min. The length of each microfiber was measured, and the shrinking ratio was calculated using Equation (1):

(1) $$\mathrm{Shrinking}\;\mathrm{ratio}\;\left(\%\right)\equiv 100\times \frac{10-L_{h}}{10}$$

where $L_{h}$ is the final length (mm) of the microfibers.

### 2.4. Shrinking Behavior of HAS Microfibers

**Figure 5:**
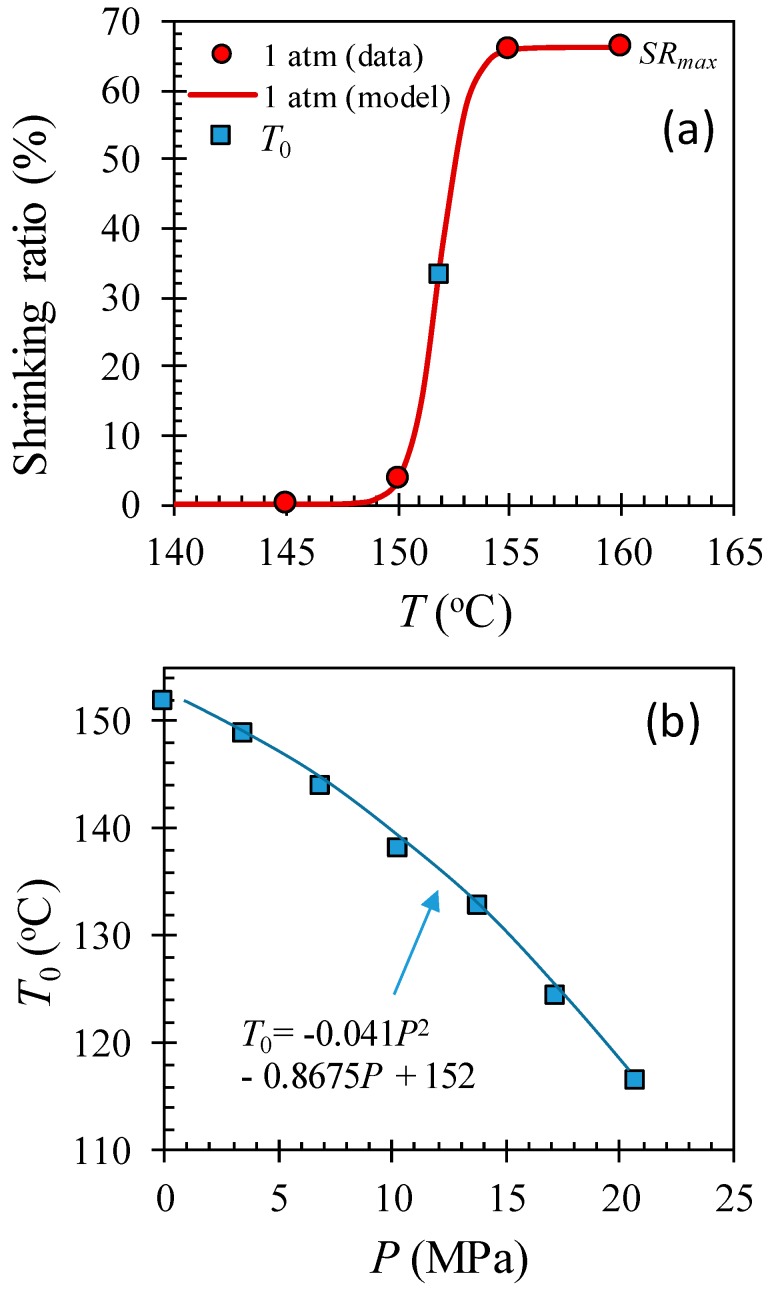
The microfiber shrinkage measurement at various temperatures and pressures in a chamber with/without CO_2_. (**a**) The shrinking ratio at 1 atm as a function of temperature and (**b**) the midpoint of shrinking ratio curve *T*_0_ as a function of CO_2_ pressure, which is decreasing with pressure, implying that the shrinking ratio curve shifts toward lower temperature with CO_2_ pressure. (Color should be used).

Figure 5 shows the shrinking ratio of HAS microfibers at temperatures between 135 and 160 °C with a 5 °C interval at various pressures with/without dissolved CO_2_. Figure 5a shows that the HAS microfibers rarely shrunk up to 150 °C, but they showed abrupt shrink activation at temperatures between 150 and 155 °C, offering a shrinking ratio of 66% at 155 °C. Such a behavior shift toward lower temperature with pressure implies that the activation temperature for shrinking decreases with CO_2_ pressure due to the plasticization effect of dissolved CO_2_ [12]. The shrinking behavior as a function of temperature and pressure can be modeled as a logistic function by Equation (2):

(2) $$\mathrm{Shrinking}\;\mathrm{ratio}\;\left(\%\right)=\frac{{\mathrm{SR}}_{\max}}{1+e^{-k\left[T-T_{0}\left(P\right)\right]}}$$

where SR_max_ is the maximum shrinking ratio in percentage, k is the steepness of the curve, and *T*_0_(P) is the temperature at the midpoint of the shrinking ratio as a function of pressure. Each parameter was found to be 66%, 1.58 °C^−1^, and 151.8 °C (at 1 atm), respectively. Figure 5b shows that *T*_0_(P) is a decreasing function of CO_2_ pressure within the experimental window, and those values can be modeled by Equation (3):

(3) $$T_{0}\left(P\right)=-0.041\;P^{2}-0.8675\;P+152$$

where *T*_0_(P) is in °C, and P is in MPa.

### 2.5. Tensile Properties of Microfibers

We hypothesized that the tensile stress in microfibers against extensional deformation fundamentally contributes to strain hardening of the matrix, and the degree of strain hardening increases with the tensile stress of the microfibers. Therefore, if there is a gap between the samples with HP and HAS microfibers from the tensile test, it would likely result in a discrepancy in the strain hardening results of the two specimens as well. Thus, obtaining the tensile properties of each microfiber would be meaningful. Our previous study [12] compared the extensional rheometry results measured with those achieved by a conventional fiber tensile test in which the sample is held between two clamps and stretched by moving one clamp away from the other at a constant speed [17,18]. Notably, extensional rheometry in a Sentmanat extensional rheometer (SER) is based on Meissner-type extension [19], where the sample length is constant during deformation, so rotating the drums that are holding the sample at a constant speed can give a constant Hencky strain rate on the sample. However, conventional tensile tests are similar to Münstedt-type [17] extension, where the sample length increases during deformation, so an exponential increase in the extension speed with time should give a constant Hencky strain rate; thus, constant speed tensile tests cannot be directly compared with data obtained using an SER.

Therefore, the microfiber tensile test was conducted with the method identical to that used for the extensional viscosity measurement (i.e., Meissner-type) using an SER fixture in a rheometer (DHR-3, TA Instruments, New Castle, DE, USA). Once the microfibers were loaded on the SER fixture at 145 °C, which is below the heat activation temperature, the tensile force reading was electronically zeroed when no tension was applied, and the temperature was increased to 155 °C. The tensile test was conducted at this temperature, at which the HAS microfibers were completely activated, i.e., fully shrunk, at a strain rate of 1 s^−1^. By controlling the temperature in this way, the HAS microfibers were activated as the tensile test was initiated.

### 2.6. Single Microfiber Pull-Out Test

To confirm adhesion between the matrix and both types of microfibers, pull-out tests [20] were performed in the tensile test fixtures in the same rheometer (DHR-3, TA Instruments, New Castle, DE, USA). Between two 3-mm-thick and 20-mm-long slabs of e-PP, a microfiber, which was longer than the slabs, was sandwiched in the center, along the long axis of the slabs. One end of the slabs was fixed vertically on one grip of the tensile fixture at 145 °C, and the microfiber was fixed on the other grip. Then, the temperature was increased to 160 °C. As soon as the temperature was set to 160 °C, the test was conducted. The microfiber was pulled out of the sample at a constant speed of 5 mm/s, which is equivalent to a shear rate of 1 s^−1^ considering the distance from the microfiber to the edge of the slabs.

### 2.7. Extensional Rheometry

Extensional rheometry measurements were performed using samples with 4 wt % microfibers, as shown in Figure 4, on the same rheometer (DHR-3, TA Instruments, New Castle, DE, USA) with an SER fixture [21,22]. The samples were tested at strain rates of 0.1, 1, and 3 s^−1^ and temperatures of 155, 160, and 165 °C. This temperature range is between the full shrinking and melting temperatures of the microfibers. Using e-PP/L-HAS and e-PP/L-HP, the measurements were carried out in the direction of alignment. The tests in the perpendicular direction were not performed because they would not show any effects of microfiber shrinkage.

### 2.8. Shear Rheometry

To determine the linear viscoelastic properties, oscillatory shear experiments were performed using the same rheometer (DHR-3, TA Instruments, New Castle, DE, USA) with 25 mm parallel plates. The storage modulus (G′) and loss modulus (G″) were determined at 155, 160, and 165 °C in a nitrogen environment. The temperatures were chosen to allow the matrix to melt while the microfibers do not, but microfiber shrinking was activated right before the measurements. Frequency sweeps from 100 to 0.01 rad/s were performed at strains within the linear viscoelastic range. A time sweep test was conducted for the same amount of time (one hour) beforehand, and the magnitude of the decrease in the complex viscosity was less than 3% in an hour, showing that thermal degradation would not be an issue for the frequency sweep tests.

### 2.9. Batch Physical Foaming

**Figure 6:**
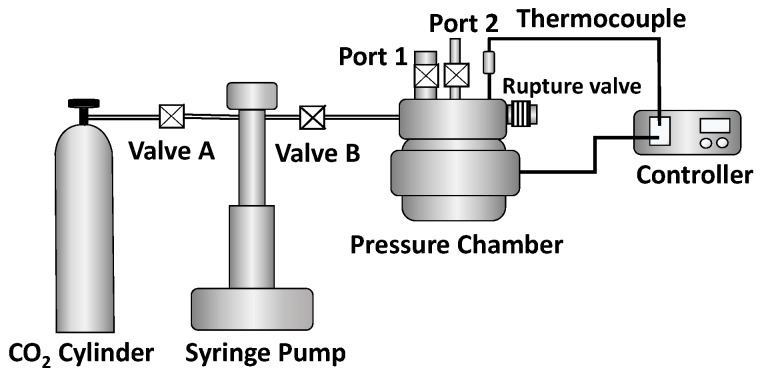
Schematic of the custom-built batch foaming system used to prepare foams of the samples in a process using CO_2_ as the foam blowing agent.

Batch physical foaming was conducted to evaluate the effects of compounded shrinking or nonshrinking microfibers on the material behavior in a real process using disk-shaped samples that were 25 mm in diameter and 3 mm in height. Figure 6 gives a schematic of the configuration of the foaming system. A chamber was heated up to set temperature between 115 and 140 °C. After placing a sample (e.g., microfibers only, e-PP, e-PP/R-HP, and e-PP/R-HAS) in the chamber, the chamber was evacuated, and CO_2_ was applied with pressures of 20.7 MPa (3000 psi) and 31 MPa (4,500 psi) for 20 min. This saturation time is estimated assuming one-dimensional CO_2_ diffusion (Figure S1) and calculating CO_2_ concentration and diffusion amount (Figures S2–S4). which was sufficient time for the e-PP matrix to be saturated according to the solution to Fick’s 2nd law of diffusion [23]. Detailed analysis is given in the supplementary material [24]. Then, the pressure was suddenly decreased at two different pressure drop rates by opening one of the two different ports to induce foaming: Port 1 for 55 MPa/s and Port 2 for 16 MPa/s.

Prior to the above foaming step, we carried out the same process for microfibers only. The plasticization effect decreases the melting point of microfibers. However, the above depressurizing tests with microfibers confirmed that microfibers did not foam in all the test conditions.

To quantify the effect of microfibers in the foam structure, the cell population density, with respect to the solid polymer, and expansion ratio were determined. After the foam morphology was observed by scanning electron microscopy (SEM, JEOL 6060), the number of bubbles was counted. Then, the cell population density was calculated by Equation (4) [25,26]:

(4) $$\mathrm{Cell}-\mathrm{population}\;\mathrm{density}\;\left(\mathrm{number}\;\mathrm{of}\;\mathrm{cells}/{\mathrm{cm}}^{3}\right)\;={\left(\frac{n}{A}\right)}^{1.5}\frac{\rho_{p}}{\rho_{f}}$$

where A is the area (cm^2^) of the SEM image, n is the number of cells in the image, and $\rho_{p}$ and $\rho_{f}$ are the densities of the unfoamed and foamed specimens, respectively. Considering the unit volume of the unfoamed polymer and not that of the foamed polymer excludes the effect of bulk volume to compare the cell population density among foams with different bulk volumes. To determine the expansion ratio, the bulk density of the foam was determined by the water displacement method according to ASTM-D792 using a density determination kit (A&D Company, AD-1653).

## 3. Results

### 3.1. Polymer Characteristic Analysis

**Figure 7:**
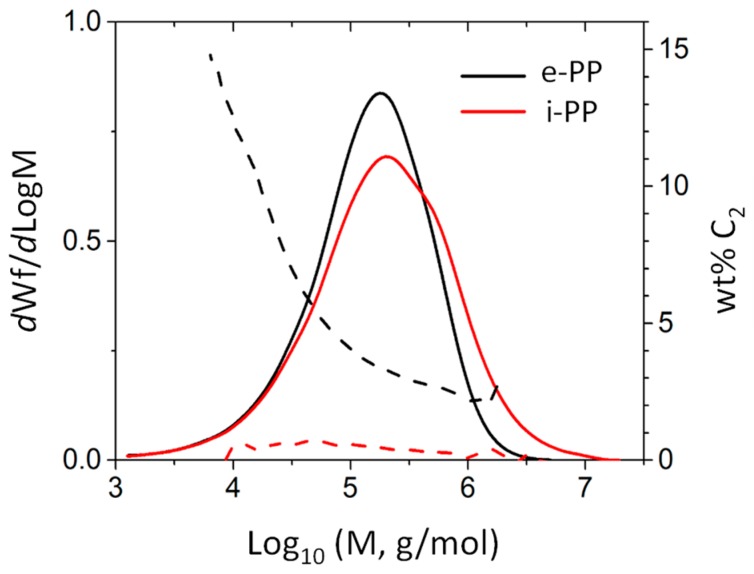
Gel permeation chromatograph (GPC) analysis result for e-PP and isotactic PP (i-PP).

To analyze the tacticity of materials, ^13^C-NMR measurements were performed at 120 °C using a 500 MHz Varian Inova NMR system (Varian Inc., Palo Alto, CA, USA) with deuterated-tetrachloroethane-d_2_ (TCE-d_2_) as the solvent. A gel permeation chromatograph (GPC, SEC-IR, Polymer Char, Valencia, Sapin) equipped with an infrared (IR) detector was used to characterize the molecular weight and comonomer content (Figure 7). A broad-band IR channel with a band region ranging from 2700 to 3000 cm^−1^ (covering all saturated C–H stretching vibrations) was used for the polymer concentration measurement, while two narrow-band channels with a central band wavenumber located at 2960 cm^−1^ (C–H in CH_3_ group) and 2920 cm^−1^ (C–H in CH_2_) were used for comonomer composition measurements. 1,2,4-trichlorobenzene (Sigma-Aldrich) with 300 ppm antioxidant (butylated hydroxytoluene) was used as the mobile phase. The molecular weight was determined by combining the universal calibration relationship with the Mark–Houwink (M–H) equation based on the M–H parameters a/K = 0.705/0.000229 for PP. The comonomer composition was determined by the ratio of the IR detector intensity corresponding to the CH_2_ and CH_3_ channels, calibrated with a series of PE and PP homo/copolymer standards whose nominal values were predetermined by NMR.

**Figure 8:**
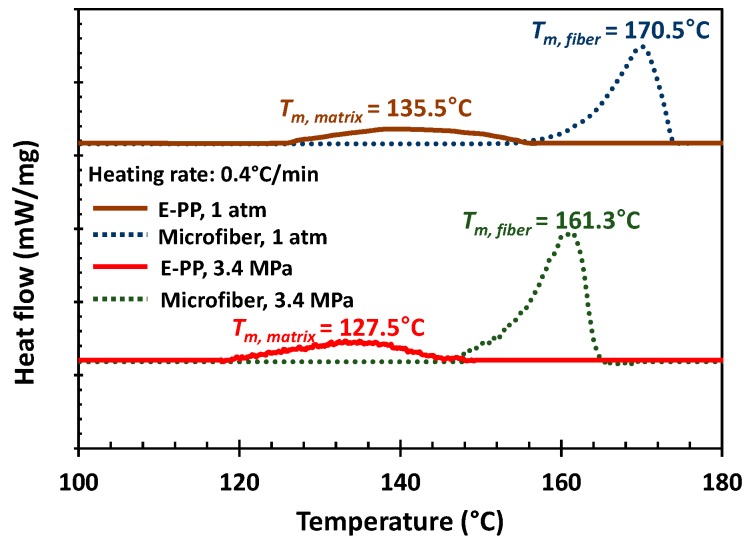
Differential scanning calorimetry (DSC) scans at heating rate of 0.4 °C/min at 1 atm and 3.4 MPa.

The melting temperatures of e-PP and microfiber (i-PP) were determined using differential scanning calorimetry (DSC, 204 HP Phoenix, Netzsch, Selb, Germany) at 1 atm and 3.4 MPa (500 psi) with CO_2_ (Figure 8), which is the maximum possible pressure to obtain reliable data. Although DSC does not cover the whole experimental window of this work, it can provide insight regarding the effect of dissolved gas on the melting point of the microfibers. A heat procedure was performed at heating rates of 0.4 °C/min, which is estimated as a value that will minimize temperature lag [27]. The peak melting points showed that e-PP had a peak melting point that was 35 °C lower than that of microfibers due to the existence of comonomer. Pressurizing with CO_2_ to 3.5 MPa reduced the melting points by 8 °C. This melting temperature drop is due to the plasticization effect of dissolved CO_2_ [12]. We chose an operating temperature window where e-PP was in the molten state while the microfibers were in the solid state, i.e., the solid microfiber could be activated for shrinking. The resulting values of the GPC, NMR, and DSC measurements are shown in Table 1. The crystallinity of polymers was determined by the area of DSC melting peak relative to the heat of fusion (165 J/g) of 100% a-crystalline isotactic polypropylene [28].

### 3.2. Tensile Test Properties of HAS and HP Microfibers

**Figure 9:**
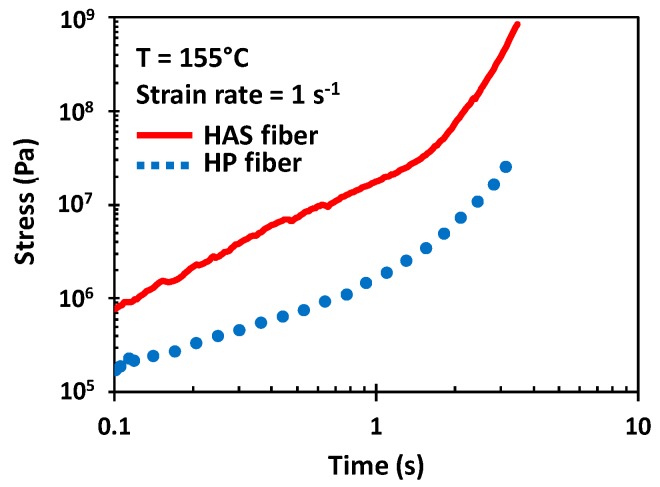
Tensile stress measurements of single HAS and HP microfibers at 1 s^−1^ using the Meissner-type test. Shrinkage of the HAS microfiber was activated in the rheometer chamber immediately preceding the beginning of the test. The results show the average of five tests. Error bars have been omitted because the standard deviation was smaller than the magnitude of data points.

Figure 9 shows the tensile stress growth function of HAS and HP microfibers at a Hencky strain rate $\dot{\varepsilon }$ of 1 s^−1^. The shrinkage of the HAS microfibers was activated due to the test temperature at the beginning of the test, which led to a tensile stress difference appearing at 0.1 s between the two types of microfibers, which was almost a difference of an order of magnitude. Furthermore, the gap increased as the test continued, and it reached a maximum of 1.5 orders of magnitude at the end of the test at 3 s when the microfibers broke. This increase in tensile strength resulted from the HAS microfiber continuously shrinking during the test, and the gap led to a difference in the strain hardening enhancement in the extensional rheometry of e-PP/HAS composites.

### 3.3. Microfiber Pull-Out Test

**Figure 10:**
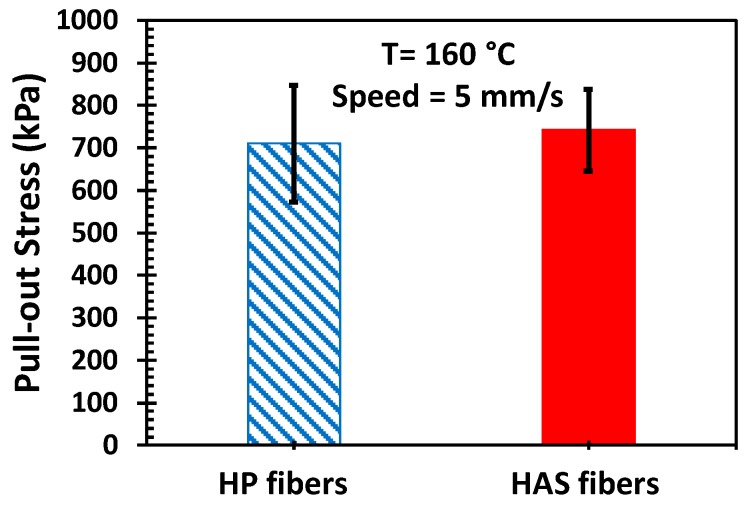
Pull-out stress of HP and HAS microfibers pulled out of the PP matrix. Error bars represent the standard deviation from five tests.

Our hypothesis was based on the assumption that the microfibers would continue to adhere to the matrix during extension; thus, the tendency to shrink would continuously exert compressive stress on the matrix and enhance strain hardening [12]. In other words, such an enhancement would not appear or would be small if there were slips between the microfibers and the matrix. However, a slip is not expected because there is a certain chemical similarity between the matrix and the microfibers, but it is important to confirm the adhesion between them. Pulling out the microfiber also pulls out a significant amount of matrix sticking to the microfiber, confirming no slip. If interfacial slip occurred, the HAS microfiber would show more slip, and the portion of the HAS microfiber in the matrix would travel faster than that of the HP microfiber due to shrinkage at a given pulling speed in which one end of both microfibers travel. This would result in more friction between the matrix and the HAS microfibers, thus causing pull-out stress. However, Figure 10 shows that the steady state values of the measured pull-out stress (pulling force/contact area) of both types of microfibers were similar, and we can infer that interfacial slip did not occur. Therefore, we can expect good adhesion between the microfibers and the matrix. Also, if any differences were to be found in the physical properties between e-PP/HP and e-PP/HAS, they would have originated from the shrinking behavior of the HAS microfibers.

### 3.4. Extensional Rheometry

**Figure 11:**
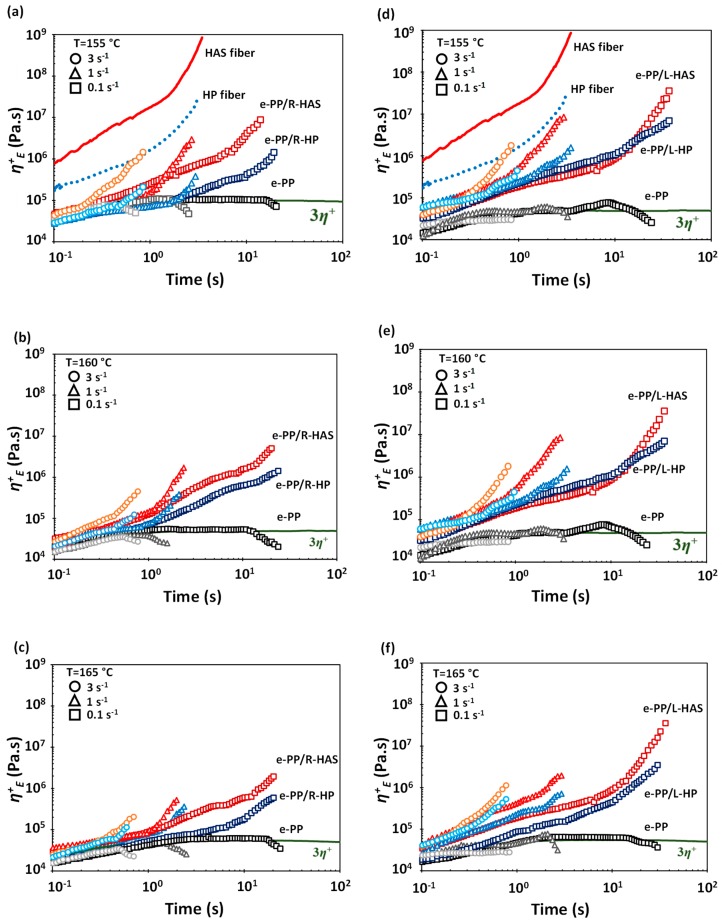
Tensile stress growth coefficient, *η*^+^*_E_*, of e-PP, HP microfibers, HAS microfibers, and composites of e-PP and microfibers at 155, 160, and 165 °C and at Hencky strain rates $\dot{\varepsilon }$ of 0.1, 1, and 3 s^−1^. The solid lines are 3 times the stress growth coefficient of steady simple shear at a strain rate of 0.05 s^−1^, which is in the linear regime; (**a**–**c**) have randomly distributed microfibers, and (**e**–**f**) have linearly aligned microfibers.

Figure 11a–c show the tensile stress growth coefficient $\eta_{E}^{+}\left(t,\dot{\varepsilon }\right)$ of neat e-PP, HAS microfibers, HP microfibers, e-PP/R-HAS, and e-PP/R-HP at various temperatures and Hencky strain rates. Figure 11d–f show the tensile stress growth coefficient $\eta_{E}^{+}\left(t,\dot{\varepsilon }\right)$ of neat e-PP, HAS microfibers, HP microfibers, e-PP/L-HAS, and e-PP/L-HP at various temperatures and Hencky strain rates. The solid line in the figures indicates the linear viscoelastic prediction of the extensional viscosity, 3η^+^(t), where η^+^(t) is the shear stress growth coefficient in the linear viscoelastic region at a shear rate of 0.05 s^−1^. The measurements were conducted at 155, 160, and 165 °C, where the shrinkage of the microfibers was heat-activated, but the microfibers did not melt, as mentioned earlier. Each test was continued until the composite ruptured. The extensional flow behavior of neat linear e-PP showed no strain hardening, as expected. While e-PP/R-HP exhibited strain hardening, e-PP/R-HAS microfibers showed an even higher degree of strain hardening. The e-PP/L-HAS and e-PP/L-HP showed generally similar trends as those of e-PP/R-HAS and e-PP/R-HP but showed a much higher degree of strain hardening and maximum *η*^+^*_E_* as well as a longer time to rupture. All these phenomena were expected as the composite was reinforced in the direction of deformation.

Figure 11a,d overlap with Figure 9, which was measured at $\dot{\varepsilon }=1{\;s}^{-1}$ and 155 °C. There was a notable difference in the maximum *η*^+^*_E_* of the composites and neat microfibers. This phenomenon was obvious, considering the small amount of compounded microfibers, but we could clearly see the origin of strain hardening (from compounding with HP or HAS microfibers) and its enhancement (from shrinking of HAS microfibers). While the neat microfibers ruptured at 3 s, e-PP/R-HP and e-PP/R-HAS ruptured at approximately 2 s. However, e-PP/L-HP and e-PP/L-HAS ruptured at the same time as the neat microfibers, which can be again attributed to the reinforced nature.

**Figure 12:**
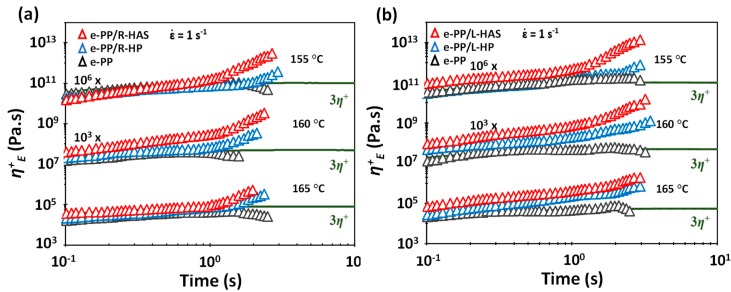
Tensile stress growth coefficient, *η*^+^*_E_*, of e-PP, e-PP/HP microfibers, and e-PP/HAS microfibers at Hencky strain rates $\dot{\varepsilon }$ of 1 s^−1^ at various temperatures (**a**) with R-HAS and R-HP and (**b**) with L-HAS and L-HP. Standard deviation bars have not been included because the ranges were smaller than the magnitude of data points.

Understanding the effect of temperature on the degree of enhancement of strain hardening is important. Figure 12 shows a comparison between e-PP/HAS, e-PP/HP, and neat e-PP specimens at the Hencky strain rate of 1 s^−1^ at three different temperatures. While the difference in the degree of strain hardening between e-PP/HP and e-PP/HAS was highest at 155 °C, it was lowest at 165 °C [12]. In other words, the difference diminished with temperature. This trend was independent of the alignment of the microfibers; the higher the temperature of the matrix, the lower was its viscosity, which resulted in easier dissipation or relaxation of the extra compressive stress from the shrinkage of HAS microfibers, and the effect of shrinkage in strain hardening was depleted. This phenomenon is important for tailoring the strain hardening using shrinking microfibers.

**Figure 13:**
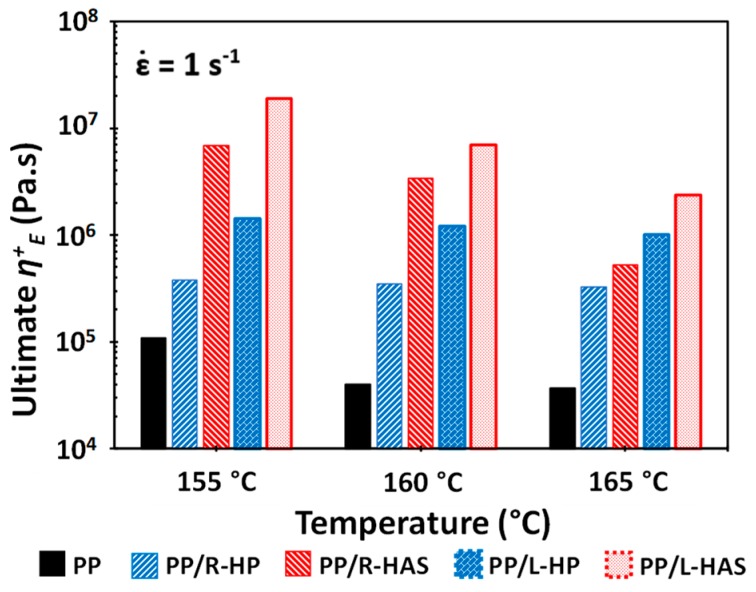
The ultimate *η*^+^*_E_* of the composites at different temperatures at a strain rate of 1 s^−1^. Error bars have been omitted because the standard deviation was small.

Figure 13 shows close comparisons of the ultimate *η*^+^*_E_*, defined as the final value of the tensile stress growth coefficient of e-PP, e-PP/HP, and e-PP/HAS microfibers at different temperatures, at the Hencky strain rate of 1 s^−1^. As expected, the tensile stress growth coefficient of e-PP decreased with temperature, while that of e-PP/R-HP did not vary with temperature. The tensile stress growth coefficient of e-PP/R-HP showed increases of 250%, 770%, and 780% over that of e-PP at 155, 160, and 165 °C, respectively. The e-PP/R-HAS composites, alternatively, showed higher values than e-PP/R-HP, yet the difference decreased with temperature (i.e., 1800%, 980%, and 160%). A similar trend was observed with composites with linearly aligned microfibers. The composite of e-PP/L-HP showed increases in the tensile stress growth coefficient of 1200%, 2900%, and 2600% over those of e-PP at 155, 160, and 165 °C, respectively. Generally, e-PP/L-HAS showed higher values than e-PP/L-HP, yet this difference again decreased with temperature (i.e., 1300%, 580%, and 230%). This trend is attributed to the melt strength of PP in which the internal stresses caused by microfiber shrinkage are dissipated in the matrix more easily, or less momentum from shrinkage is transferred to the matrix as the temperature increases [12]. Notably, the ultimate *η^+^_E_* of e-PP/HP was less affected by temperature. The dependence and independence of temperature have important roles in controlling strain hardening. These trends result because the lower viscosity of the matrix at higher temperatures causes the compressive stress from the HAS microfibers to easily dissipate; thus shrinkage provides fewer advantages. This phenomenon can be exploited to tune the behaviors and properties in applications such as foaming by controlling the processing temperatures when HAS microfibers are compounded and the degree of shrinking in engineered microfibers.

Because the shrinkage of the microfibers occurs in longitudinal direction (i.e., the direction considered to be lengthwise), the specimens with linearly aligned microfibers showed higher extensional stress than those with randomly distributed microfibers. At all temperature conditions, both e-PP/L-HAS and e-PP/L-HP showed 2–4 times higher ultimate values compared to e-PP/R-HAS and e-PP/R-HP. From this result, it is expected that aligning microfibers in the matrix would be beneficial in processes with unidirectional flow, such as fiber spinning.

### 3.5. Linear Viscoelastic Shear Behavior

**Figure 14:**
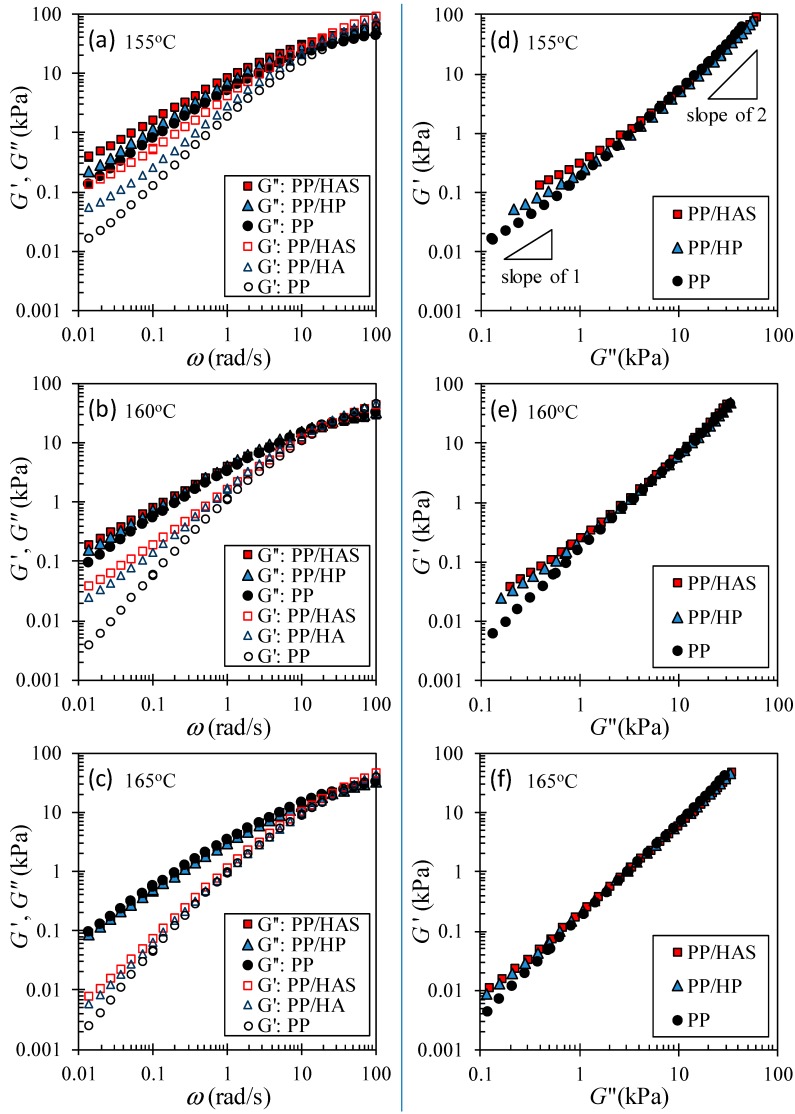
Linear viscoelastic behavior of neat PP, melt-compounded PP/HP microfibers (96/4 wt %), and PP/HAS microfibers (96/4 wt %) frequency dependence of the storage (G’) and loss (G”) moduli for the three samples at (**a**,**d**) 155 °C, (**b**,**e**) 160 °C, and (**c**,**f**) 165 °C. (Color should be used).

A microfiber network was detected by measuring the storage (G’) and loss (G”) moduli as a function of frequency (ω). Figure 14a–c show that both moduli of e-PP/R-HP and e-PP/R-HAS had upturns over e-PP, especially at low frequencies. We attribute this shift to the presence of solid microfibers that did not melt at the test temperature and that reduced the stress relaxation behavior of the matrix on a universal scale rather than on a local scale of the molecules. This effect was more pronounced in G’ than G” in the logarithmic scale, i.e., the effect of compounding with microfibers was more apparent in elastic properties than in viscous properties, which can be seen more clearly in Han plots [29]. Figure 14d–f show that the existence of the microfiber, and the shrinking nature caused increases at low frequencies (to the left and bottom). The e-PP/R-HAS composite showed even higher moduli compared to e-PP/R-HP. This increase is attributed to the shrinking behavior of HAS, which not only remained in the matrix in solid form but also actively restricted the motion of the matrix and prevented relaxation of the matrix by exerting internal stress.

As the temperature increased, the difference in moduli between e-PP/R-HP and ePP/R-HAS decreased. This trend, similar to that of extensional rheometry, is attributed to the easier dissipation of internal stresses in the matrix caused by microfiber shrinkage or the transfer of less momentum from shrinkage to the matrix as the temperature increases [12]. The effect of the microfibers is also similar to the cylindrical structure in block copolymers with phase separation. Kossuth et al. [30] showed that the slope of G’(ω) is 1/3 that at low frequencies below the phase separation temperature. Therefore, studies with microfibers and block copolymers can be models for researching each other.

### 3.6. Batch Foaming

Batch physical foaming was conducted with e-PP, e-PP/R-HAS, and e-PP/R-HP. The foaming process of the microfiber itself was also conducted under identical conditions, and it was experimentally confirmed that the microfiber does not foam; this is important because, if they are foamed, a decreased effect of the microfibers and shrinkage would be expected. Also, under identical conditions, it was found that the HAS microfibers shrunk at 115 °C or higher temperatures. Therefore, in order to investigate the shrinking behavior of microfibers, the foaming test was conducted at no lower than 115 °C.

**Figure 15:**
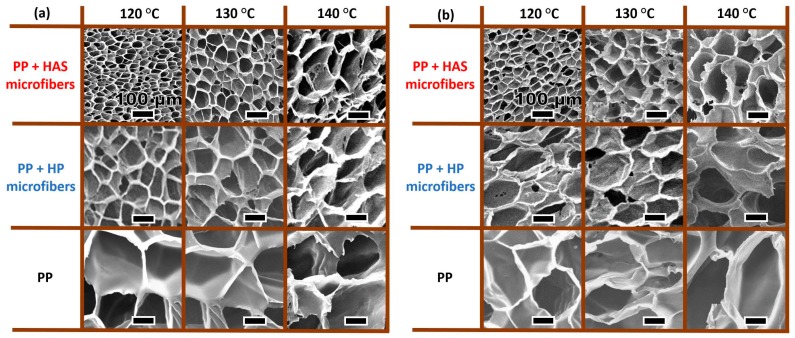
SEM micrographs of foams obtained after batch foaming processes (**a**) at 31.0 MPa and (**b**) at 20.7 MPa.

**Figure 16:**
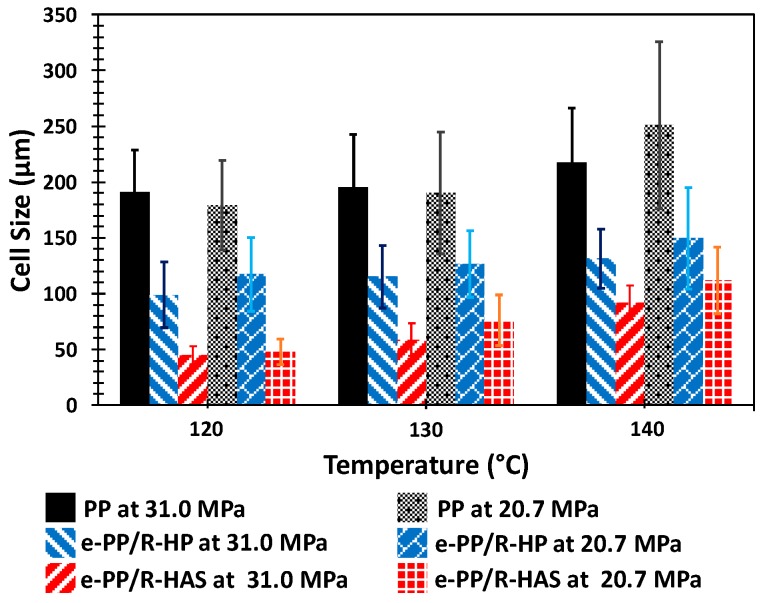
Cell size analysis for the samples from batch foaming process.

Figure 15 shows the cell structure of the three samples (i.e., e-PP, e-PP/R-HP, and e-PP/R-HAS specimens) at three different processing temperatures (120, 130, and 140 °C) and two different pressures and pressure drop rates (31 MPa/55 MPa/s and 20.7 MPa/16 MPa/s). As shown in Figure 16, in the order of e- PP < e-PP/R-HP < e-PP/R-HAS and 140 °C < 130 °C < 120 °C, the cell size decreased and each foam structure became more uniform.

**Figure 17:**
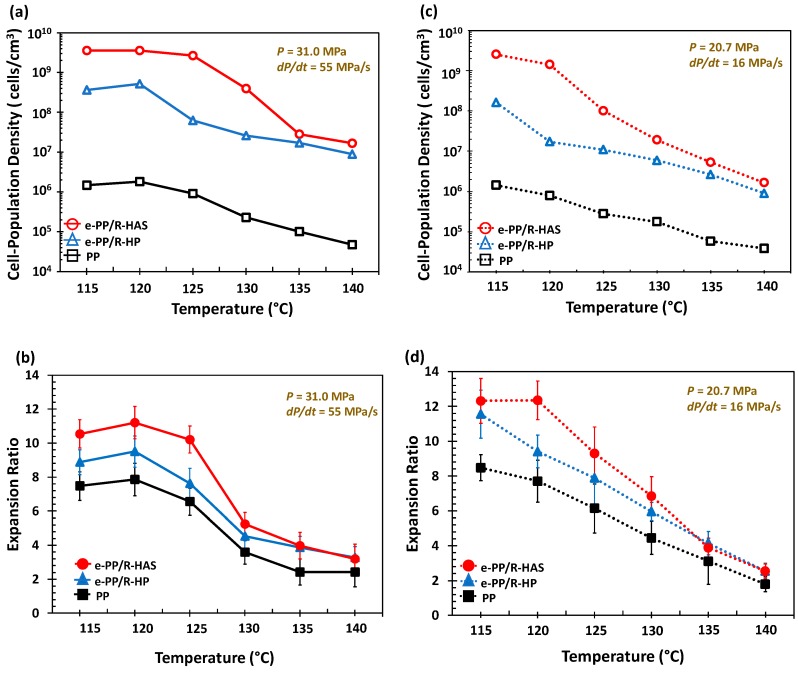
Characterization of the cell population and foam densities measured after batch foaming at various temperatures at two pressures and pressure drop rates. (**a**) Cell population density at 31.0 MPa, (**b**) the expansion ratio at 31.0 MPa, (**c**) cell population density at 20.7 MPa, (**d**) the expansion ratio at 20.7 MPa. Standard deviation bars have not been included in (**a**, **b**) because they were smaller than the data points. Lines have been added only as a guide and are not a model prediction. The results show the average value of five measurements. Error bars in (**a**, **b**) have been omitted because the standard deviation was smaller than the magnitude of data points.

Figure 17 shows the cell population density and expansion ratio over the studied temperature range (115–140 °C) and pressure conditions (31 MPa/55 MPa/s and 20.7 MPa/16 MPa/s). The cell population density of e-PP ranged from 10^4^ to 10^6^ cells/cm^3^, the e-PP/R-HP microfiber samples ranged from 10^7^ to 10^9^ cells/cm^3^, and the e-PP/R-HAS microfiber samples ranged from 10^7^ to 10^10^ cells/cm^3^. The expansion ratio of e-PP ranged from 2 to 8, those of the e-PP/R-HP microfiber samples ranged from 3 to 11, and those of the e-PP/R-HAS microfiber samples ranged from 3 to 12. E-PP/R-HP microfibers showed higher cell population densities and expansion ratios than neat e-PP due to the presence of microfibers, which induced stress variations, thus decreasing cell coalescence. This effect was enhanced further with e-PP/R-HAS microfibers. The increase of cell population density with HAS microfibers was much more significant than that with macrosized HAS fibers [12] (i.e., approximately 4200% of magnitude increase in cell population density in this study compared to 450% of increase in our previous study). The degree of improvement was highest at 120 °C and decreased as the processing temperature increased, which is consistent with the extensional viscosity measurement result in Figure 13 as well as the linear viscoelastic behavior in Figure 14 in which a low melt strength of the matrix at a high temperature caused the dissipation of internal stresses formed by shrinkage of the microfibers. A high expansion ratio indicates that a large amount of gas is retained within the closed cells of the foam, and gas escaping from the polymer is inhibited [2]. A decrease in the viscosity of the matrix due to an increase in temperature caused greater dissipation of the compressive stress formed by heat activation of the HAS microfibers; thus, the positive effect of microfibers on foaming decreased with temperature. This effect was more prominent with HAS microfibers than with HP microfibers, implying that cell population density and foam density of foams with microfibers can be tailored by controlling processing conditions, such as temperature with shrinking microfibers, and a wider range of cell/foam density can be expected than with passive microfibers.

## 4. Conclusions

We proved that compounding microfibers that have active-shrinking behavior into thermoplastic polymer is a feasible approach to enhance and control strain hardening and thus foamability. The results demonstrate that improvement and control of strain hardening of PP by compounding in situ shrinking microfibers are clear. It was demonstrated that the degree of in situ shrinkage can control the strain hardening behavior and the temperature dependence of composite structures. Yet, this trend is independent of the microfiber orientation (i.e., linearly aligned and randomly distributed). An application of the strain hardening control was also confirmed in batch physical foaming. This technology may be able to provide an alternative solution for typically nonfoamable resins to be utilized to produce superior foam structures without long-chain branching and/or cross-linking. This strategy is expected to be applicable not only to foaming process but also to various polymers with no foaming ability to control the viscoelastic properties.

Subsequent research studies will include the effects of microfiber size, aspect ratio, more specified microfiber orientation, processing conditions during microfiber generation, and degree of in situ shrinking on rheology and foam morphology because they are key variables in our smart microfiber blending technology. In addition, further studies will be conducted with nanoscale shrinking microfibers to more significantly enhance strain hardening and foamability.

## Figures and Tables

**Table 1:** Polymer characteristics.

Code	Function	Molecular Weights (kg/mol)			*M*_w_/*M*_n_	Tacticity (Triad mol %)			Comonomer Content		Peak *T*_m_ (°C)		Crystallinity (%)
		*M*_n_	*M*_w_	*M*_z_		rr	mm	mr	mol %	mass %	1 atm	3.4 MPa with CO_2_	
e-PP	matrix	55	252	621	4.58		^a^ 84.7		propylene (92) ethylene (8)	propylene (94.5) ethylene (5.5)	135.5	127.5	14.5
i-PP	micro- fiber	60	451	2080	7.55	3	93	4	^b^ N.A.	^b^ N.A.	170.5	161.3	^c^ 62.8

^a^ % mm from PP and EP sequences: mm from PP and EP/(mm + mr + mr) from PP and EP. ^b^ i-PP is a homopolymer. ^c^ HAS microfibers.

## Supplementary Materials

The following are available online at http://www.mdpi.com/2073-4360/11/2/211/s1, Figure S1. CO_2_ diffusion in x-direction. Figure S2. Relative concentration in the center of the polymer sheet. Figure S3. Relative quantity absorbed by the polymer sheet. Figure S4. Relative quantity absorbed by the polymer sheet.

## References

[1] Gabriel C., Münstedt H. (2003). Strain Hardening of Various Polyolefins in Uniaxial Elongational Flow. J. Rheol..

[2] Rizivi A., Andalib Z.K.M., Park C.B. (2017). Fiber-spun Polypropylene/Polyethylene Terephthalate Microfibrillar Composites with Enhanced Tensile and Rheological Properties and Foaming Ability. Polymer.

[3] Spitael P., Macosko C.W. (2004). Strain Hardening in Polypropylenes and Its Role in Extrusion Foaming. Polym. Eng. Sci..

[4] Xu Z., Zhang Z., Guan Y., Wei D., Zheng A. (2013). Investigation of Extensional Rheological Behaviors of Polypropylene for Foaming. J. Cell. Plast..

[5] Zhang Y., Parent J.S., Kontopoulou M., Park C.B. (2015). Foaming of Reactively Modified Polypropylene: Effects of Rheology and Coagent Type. J. Cell. Plast..

[6] Stange J., Münstedt H. (2006). Effect of Long-chain Branching on the Foaming of Polypropylene with Azodicarbonamide. J. Cell. Plast..

[7] Torres E., Li S., Costeux S., Dealy J.M. (2015). Branching Structure and Strain Hardening of Branched Metallocene Polyethylenes. J. Rheol..

[8] Tabatabaei S.H., Carreau P.J., Ajji A. (2010). Rheological Properties of Blends of Linear and Long-chain Branched Polypropylenes. Polym. Eng. Sci..

[9] Jahani Y., Ghetmiri M., Vaseghi M.R. (2015). The Effects of Long Chain Branching of Polypropylene and Chain Extension of Poly(ethylene terephthalate) on the Thermal Behavior, Rheology and Morphology of Their Blends. RSC Adv..

[10] Ameli A., Jung P.U., Park C.B. (2013). Electrical Properties and Electromagnetic Interference Shielding Effectiveness of Polypropylene/carbon Fiber Composite Foams. Carbon.

[11] Zhou Y., Wu W., Zou J., Turng L. (2016). Dual-scale Modeling and Simulation of Film Casting of Isotactic Polypropylene. J. Plast. Film. Sheet..

[12] Kim E.S., Park H.E., Lee P.C. (2018). In situ Shrinking Fibers Enhance Strain Hardening and Foamability of Linear Polymers. Polymer.

[13] Maddah H.A. (2016). Polypropylene as a Promising Plastic: A review. Am. J. Polym. Sci..

[14] Drabek J., Zatloukal M. (2016). Evaluation of Thermally Induced Degradation of Branched Polypropylene by Using Rheology and Different Constitive Equations. Polymers.

[15] Mohebbi A., Mighri F., Ajji A., Rodrigue D. (2015). Current Issues and Challenges in Polypropylene Foaming: A Review. Cell. Polym..

[16] Yang Y., Boom R., Heerden D.V., Kuiper P., Wit H.D. (2012). Recycling of Composite Materials. Chem. Eng. Process..

[17] Münstedt H. (1979). New Univeral Extensional Rheometer for Polymer Melts. Measurements on a Polystyrene Sample. J. Rheol..

[18] Bach A., Rasmussen H.K., Hassager O. (2003). Extensional Viscosity for Polymer Melts Measured in the Filament Stretching Rheometer. J. Rheol..

[19] Meissner J., Hostettler J. (1994). A New Elongational Rheometer for Polymer Melts and Other Highly Viscoelastic Liquids. Rheol. Acta..

[20] Kim J., Mai Y. (1998). Engineered Interfaces in Fiber Reinforced Composites.

[21] Baired D.G., Collias D.I. (1993). Polymer Processing: Principles and Design.

[22] Dealy J.M., Morris J., Morrison F., Vlassopoulos D. (2013). Official symbols and nomenclature of The Society of Rheology. J. Rheol..

[23] Sato Y., Fujiwara K., Takikawa T., Takishima S., Masuoka H. (1999). Solubilities and Diffusion Coefficients of Carbon Dioxide and Nitrogen in Polypropylene, High-density Polyethylene, and Polystyrene under Higher Pressures and Temperatures. Fluid Phase Equilibr..

[24] Park H.E. (2005). Effects of Pressure and Dissolved Carbon Dioxide on the Rheological Properties of Molten Polymers. Ph.D. Thesis.

[25] Wang L., Lee R., Wang G., Chu R.K.M., Zhao J., Park C.B. (2017). Use of Stereocomplex Crystallites for Fully-biobased Microcellular Low-density Poly(lactic acid) Foams for Green Packaging. Chem. Eng. J..

[26] Tran M., Gong P., Detrembleur C., Thomassin J., Buahom P., Saniei M., Kenig S., Park C.B., Lee S. (2016). Reducing Thermal Conductivity of Polymeric Foams with High Volume Expansion Made From Polystyrene/expanded Graphite. SPE ANTEC Indianap..

[27] Carslaw H.S., Jaeger J.C. (1986). Conduction of Heat in Solids.

[28] Wunderlich B. (1980). Macromolecular Physics. Vol. 3: Crystal Melting.

[29] Han C.D., Baek D.M., Kim J.K., Ogawa T., Sakamoto N., Hashimoto T. (1995). Effect of Volume Fraction on Order-Disorder Transition in Low Molecular Weight Polystyrene-block-Polyisoprene Copolymers. Macromolecules.

[30] Kossuth M.B., Morse D.C., Bates F.S. (1999). Viscoelastic Behavior of Cubic Phases in Block Copolymer Melts. J. Rheol..

