# Supplementary material for: Enhanced Foamability with Shrinking Microfibers in Linear Polymer
Source: Polymers (Basel). 2019 Jan 26;11(2):211. doi: 10.3390/polym11020211 (PMC6419035; doi:10.3390/polym11020211)
Supplement: Supplementary file 1 [file polymers-11-00211-s001.pdf]

polymers

MDPI

Supplementary Material

Enhanced Foamability with Shrinking Microfibers in Linear Polymer

Eric S. Kim [1], Heon E. Park [2,*], Carlos R. Lopez-Barron [3], Patrick C. Lee [1,*]

[1] Department of Mechanical and Industrial Engineering, University of Toronto, 5 King's College Road, Toronto, Ontario M5S 3G8, Canada; ericskim@mie.utoronto.ca (E.K.); patricklee@mie.utoronto.ca (P.L.)
[2] Department of Mechanical Engineering, University of Vermont, 33 Colchester Avenue, Burlington, Vermont, 05405, USA; heon.e.park@gmail.com
[3] ExxonMobil Chemical Company, Baytown Technology and Engineering Complex, Baytown, Texas 77520, USA; carlos.r.lopez-barron@exxonmobil.com

* Correspondence: patricklee@mie.utoronto.ca (P.L.); heon.e.park@gmail.com (H.P.)

1. Fick's law of diffusion

Fick's first law states that the flux is proportional to the concentration gradient at steady state:

$$\vec{F} = -D\nabla \mathrm{C} \tag{1}$$

Where $\vec{F}$ denotes the flux vector that gives the amount of substance diffusion across a unit area in unit time, C is the concentration of diffusing substance, and D is the diffusion coefficient. The x-component of the flux vector can be expressed as:

$$\mathrm{F_x} = -D\left(\frac{\delta C}{\delta x}\right) \tag{2}$$

Fick's second law describes unsteady-state diffusion:

$$\frac{\delta C}{\delta t} = -\left(\frac{\delta F_x}{\delta x} + \frac{\delta F_y}{\delta y} + \frac{\delta F_z}{\delta z}\right) = \frac{\delta(D\delta C)}{\delta x^2} + \frac{\delta(D\delta C)}{\delta y^2} + \frac{\delta(D\delta C)}{\delta z^2} \tag{3}$$

Where C indicates a function of x, y, z, and t. If D is independent of C, Equation (3) can be expressed as,

$$\frac{\delta C(x,y,z,t)}{\delta t} = D\left[\frac{\delta^2 C(x,y,z,t)}{\delta x^2} + \frac{\delta^2 C(x,y,z,t)}{\delta y^2} + \frac{\delta^2 C(x,y,z,t)}{\delta z^2}\right] \tag{4}$$

1.1. Solution of the 1-D Fick's equation

The diffusion is assumed to take place only in the x-direction, as shown in Figure S1. Then, Equation 4 can be simplified to,

$$\frac{\delta C(x,t)}{\delta t} = D\left[\frac{\delta^2 C(x,t)}{\delta x^2}\right] \tag{5}$$

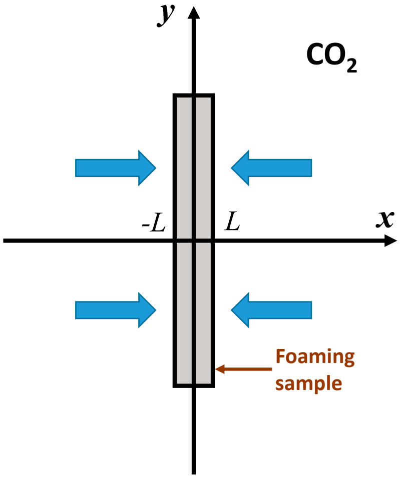

Figure S1. CO2 diffusion in x-direction.

The region –*L* < *x* < *L* is initially at a uniform concentration: $C(x,0) = C_0$. There is no net diffusion in the center: $\delta C(x)/\delta x = 0$ at $x = 0$, and the surfaces are kept at a constant concentration: $C = [\pm L(t), t] = C_1$. Using the initial and boundary conditions, the concentration at any point in the sample at time *t* is given by Equation (6),

$$\frac{C(x,t) - C_1}{C_0 - C_1} = \frac{4}{\pi} \sum_{n=0}^{\infty} \frac{(-1)^n}{2n+1} \exp\left[\frac{-D(2n+1)^2 \pi^2 t}{4L^2}\right] \cos\frac{(2n+1)\pi x}{2L} \tag{6}$$

In order to make it as a form that the degree of saturation is obtained, the equation can be rearranged as,

$$\frac{C(x,t) - C_0}{C_1 - C_0} = 1 - \frac{4}{\pi} \sum_{n=0}^{\infty} \frac{(-1)^n}{2n+1} \exp\left[\frac{-D(2n+1)^2 \pi^2 t}{4L^2}\right] \cos\frac{(2n+1)\pi x}{2L} \tag{7}$$

This quantity has the following properties:

$$\lim_{t \to 0} \frac{C(x,t) - C_0}{C_1 - C_0} = 0 \tag{8}$$

$$\lim_{t \to \infty} \frac{C(x,t) - C_0}{C_1 - C_0} = 1 \tag{9}$$

The concentration at *t=0* is zero. Thus, C_0 is zero, and C_1 is the solubility S of the diffusant in the polymer assuming instant local equilibrium at the interface. Then Equation (7) reduces to,

$$\frac{C(x,t)}{S} = 1 - \frac{4}{\pi} \sum_{n=0}^{\infty} \frac{(-1)^n}{2n+1} \exp\left[\frac{-D(2n+1)^2 \pi^2 t}{4L^2}\right] \cos\frac{(2n+1)\pi x}{2L} \tag{10}$$

The concentration in the center of the sample, $C(0,t)$ can be used to estimate the saturation time, because the center will be saturated last:

$$\frac{C(0,t)}{S} = 1 - \frac{4}{\pi} \sum_{n=0}^{\infty} \frac{(-1)^n}{2n+1} \exp\left[\frac{-D(2n+1)^2 \pi^2 t}{4L^2}\right] \tag{11}$$

The quantity $C(0,t)/S$ is a measure of the level of saturation at time *t*. If $M(t)$ is the total amount of diffusing substance absorbed by the sheet at time *t*, and $M(\infty)$ is the corresponding quantity after

infinite time, these quantities can be obtained by integrating the concentration over the width of follows:

$$M(t) \equiv \int_{-L}^{L} C(x,t)\,dx = 2\int_{0}^{L} C(x,t)\,dx$$

$$= 2S\int_{0}^{L}\left\{1 - \frac{4}{\pi}\sum_{n=0}^{\infty}\frac{(-1)^n}{2n+1}\exp\left[-\frac{D(2n+1)^2\pi^2 t}{4L^2}\right]\cos\frac{(2n+1)\pi x}{2L}\right\}dx$$

$$= 2SL - 2SL\sum_{n=0}^{\infty}\frac{8}{(2n+1)^2\pi^2}\exp\left[-\frac{D(2n+1)^2\pi^2 t}{4L^2}\right] \quad (12)$$

$$M(\infty) = \lim_{t\to\infty} M(t) = 2SL \quad (13)$$

The quantity $M(t)/M(\infty)$ is another measure of the level of saturation at time t and is given by Equation 14,

$$\frac{M(t)}{M(\infty)} = 1 - \sum_{n=0}^{\infty}\frac{8}{(2n+1)^2\pi^2}\exp\left[\frac{-D(2n+1)^2\pi^2 t}{4L^2}\right] \quad (14)$$

Thus, the time required to reach a given saturation level in terms of $C(0,t)$ or $M(t)$ can be estimated if D is known. The solutions of Equations (11) and (14) can be represented by plots of $C(0,t)/S$ or $M(t)/M(\infty)$ versus dimensionless diffusion time, Dt/L^2, and this plot can be applied with samples having any vales of *D*, *a*, and *L*. From this chart, the time for a certain level of saturation can be easily calculated with given *D*, *a*, and *L*. Figures S3 show this plot.

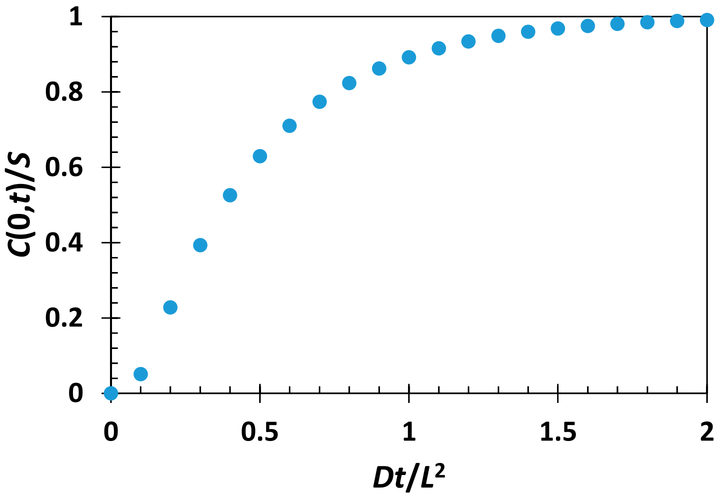

Figure S2. Relative concentration in the center of the polymer sheet.

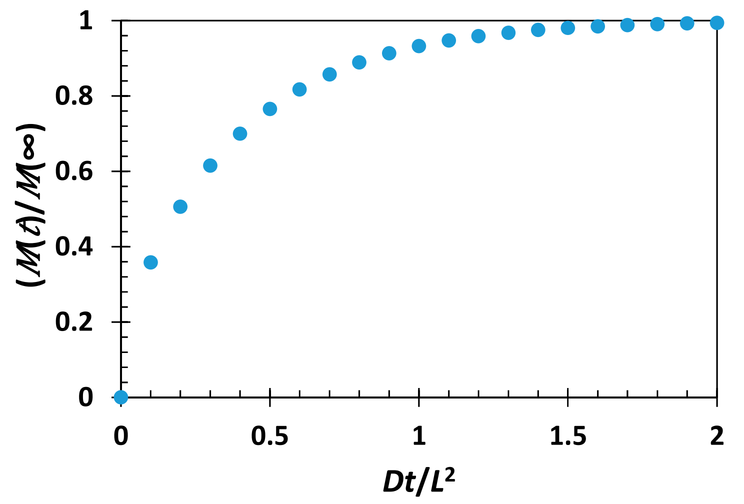

Figure S3. Relative quantity absorbed by the polymer sheet.

1.2. Moving boundary

The edges of the sample actually move outward with time due to swelling, and L thus increases with t. Equation (5) with moving boundaries will predict a longer saturation time than that with fixed boundaries. A moving boundary problem can be estimated from solutions to problems with an unswollen fixed boundary and a totally swollen, fixed boundary. The degree of swelling was estimated from the study conducted by Lei at el.,[1] which was 1.05. Therefore, the linear swell occurs at $\sqrt[3]{1.05} \approx 1.016$. This would not bring significant difference to the swelling time estimation. Thus, the solution to the unswollen problem was used for this study.

1.3. Estimate of the saturation time

From the experiment studied by Sato et al.,[2] D for the PP was estimated to around 1.47×10^{-8} (m^2/s) at our test conditions (i.e., the temperature of 115 – 140°C and the pressure of 3000, 4500 psi). The thickness of sample is 3 mm, so L (half the sample thickness) is 1.5 mm. Figure S4 shows that Dt/L^2 is 1.77 for $M(t)/M(\infty)$=0.99 and 1.97 for $C(0,t)/S$=0.99. The corresponding times for 99% saturation are then 4.5 and 5.0 min, respectively. Therefore, the 20 min is sufficient time for the e-PP matrix to be saturated according to the solution to the Fick's 2nd law of diffusion.

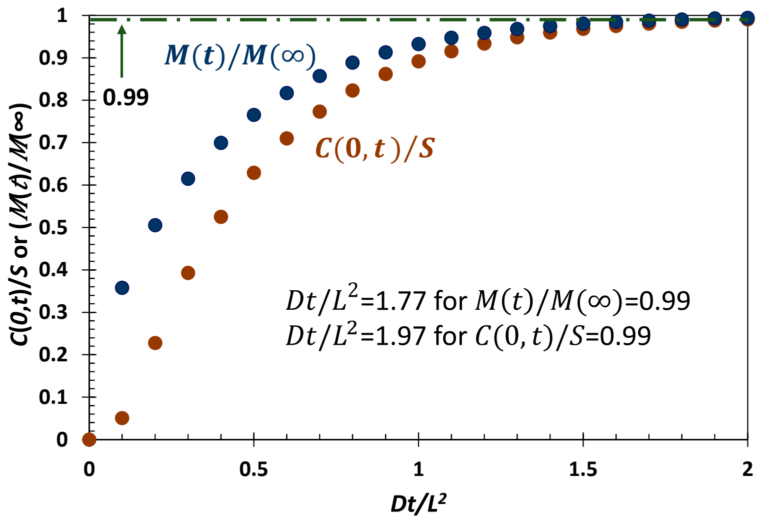

Figure S4. Relative quantity absorbed by the polymer sheet.

References

1 Lei, Z.; Ohyabu, H.; Sato, Y.; Inomata, H.; Smith Jr., R.L. Solubility, Swelling Degree and Crystallinity of Carbon Dioxide-Polypropylene System. *J. Supercrit. Fluids* **2007**, 40, 452-461, https://doi.org/10.1016/j.supflu.2006.07.016

2 Sato, Y.; Fujiwara, K.; Takikawa, T.; Sumarno; Takishima, S.; Masuoka, H. Solubilities and Diffusion coefficients of carbon dioxide and nitrogen in polypropylene, high-density polyethylene, and polystyrene under high pressures and temperatures. *Fluid Phase Equilib.* **1999** 162, 261-276, https://doi.org/10.1016/S0378-3812(99)00217-4
